# Automated cortical auditory evoked potentials threshold estimation in neonates^☆^

**DOI:** 10.1016/j.bjorl.2018.01.001

**Published:** 2018-02-02

**Authors:** Lilian Sanches Oliveira, Dayane Domeneghini Didoné, Alessandra Spada Durante

**Affiliations:** a Faculdade de Ciências Médicas da Santa Casa de São Paulo, Escola de Fonoaudiologia e Audiologia, São Paulo, SP, Brazil; b Universidade Federal do Rio Grande do Sul, Programa de Pós-graduação em Saúde da Criança e do Adolescente, Rio Grande do Sul, RS, Brazil

**Keywords:** Audiology, Auditory evoked potentials, Infant, newborn, Electrophysiology, Audiologia, Potenciais evocados auditivos, Lactente, recém-nascido, Eletrofisiologia

## Abstract

**Introduction:**

The evaluation of cortical auditory evoked potential has been the focus of scientific studies in infants. Some authors have reported that automated response detection is effective in exploring these potentials in infants, but few have reported their efficacy in the search for thresholds.

**Objective:**

To analyze the latency, amplitude and thresholds of cortical auditory evoked potential using an automatic response detection device in a neonatal population.

**Methods:**

This is a cross-sectional, observational study. Cortical auditory evoked potentials were recorded in response to pure-tone stimuli of the frequencies 500, 1000, 2000 and 4000 Hz presented in an intensity range between 0 and 80 dB HL using a single channel recording. P1 was performed in an exclusively automated fashion, using Hotelling's *T*^2^ statistical test. The latency and amplitude were obtained manually by three examiners. The study comprised 39 neonates up to 28 days old of both sexes with presence of otoacoustic emissions and no risk factors for hearing loss.

**Results:**

With the protocol used, cortical auditory evoked potential responses were detected in all subjects at high intensity and thresholds. The mean thresholds were 24.8 ± 10.4 dB NA, 25 ± 9.0 dB NA, 28 ± 7.8 dB NA and 29.4 ± 6.6 dB HL for 500, 1000, 2000 and 4000 Hz, respectively.

**Conclusion:**

Reliable responses were obtained in the assessment of cortical auditory potentials in the neonates assessed with a device for automatic response detection.

## Resumo

**Introdução:**

O potencial evocado auditivo cortical tem sido o foco de estudos científicos. Alguns autores observaram que a detecção automatizada de respostas é eficaz na exploração desses potenciais em lactentes, mas poucos relataram sua eficácia na busca de limiares.

**Objetivo:**

Analisar a latência, a amplitude e os limiares do potencial evocado auditivo cortical em recém-nascidos, com o uso de um dispositivo de detecção automática de resposta.

**Método:**

Estudo transversal, observacional. Os potenciais evocados auditivos corticais foram registrados em resposta a estímulos de tons puros nas frequências de 500, 1000, 2000 e 4000 Hz e apresentados em uma faixa de intensidade entre 0–80 dBNA, com o uso de gravação de canal único. O P1 foi feito de forma exclusivamente automática, com o uso do teste estatístico *T*^2^ de Hotelling. A latência e a amplitude foram obtidas manualmente por três examinadores. O estudo incluiu 39 recém-nascidos com até 28 dias de idade de ambos os sexos, com presença de emissões otoacústicas e sem fatores de risco para perda auditiva.

**Resultados:**

Com o protocolo usado, as respostas dos PEAC foram detectadas em todos os indivíduos em alta intensidade e limiares. Os limiares médios foram 24,8 ± 10,4 dBNA, 25 ± 9,0 dBNA, 28 ± 7,8 dBNA e 29,4 ± 6,6 dBNA para 500, 1000, 2000 e 4000 Hz, respectivamente.

**Conclusão:**

Foram obtidas respostas confiáveis na avaliação dos potenciais auditivos corticais em recém-nascidos com um dispositivo para detecção de resposta automática.

## Introduction

Hearing is a key function supporting the communication process among individuals. It is through hearing that a child can experience the world of sound, promoting the development of spoken language. The anatomical and functional integrity of the peripheral and central auditory system, together with exposure to auditory experiences, are basic requisites for the normal acquisition and development of language.^1^ Thus, assuring reliable estimates of auditory thresholds in the infants is paramount. However, accurately investigating hearing in the first months of life has always posed a challenge for audiologists.

In the first months of life, it is not possible to accurately determine auditory thresholds by observation of behavioral responses to sound stimuli alone. Therefore, objective measures of hearing are essential tools to properly verify subjective observations and integrate this cross-check investigation. One important objective measure is Auditory Evoked Potential (AEP). AEP results from neural activities in the auditory pathways in response to a sound stimulus and some types of AEP are useful for establishing auditory thresholds in infants.^2^

Neural electrical activity generated by acoustic stimuli can be detected at many different levels of the auditory pathway, according to the response latency in relation to the stimuli (milliseconds – ms). These responses can be classified into three groups: short latency AEP, i.e. those occurring within the first 10–12 ms; medium latency AEP, generated within 12–50 ms, and long latency (or cortical) AEP which occur at 50–600 ms.^3^

Cortical Auditory Evoked Potentials (CAEP) were discovered in the 1930s and thoroughly researched in the 1960s and 1970s.

The CAEP are represented, in adults, by a complex of waves called P1, N1 and P2. In normal hearing neonates, CAEP response occurs at a marked positive peak at around 200–300 ms after acoustic stimulus,^4^ with changes in form and latency of components taking place during the first 14–16 years of life. Morphological changes and maturation of the Central Nervous System (CNS) improve synaptic effectiveness and are responsible for these shifts in response latencies during the first years of life.^5^, ^6^

The detection of CAEP has numerous benefits, including the ability to assess the whole auditory system, i.e. from the brainstem to the auditory cortex. Another advantage is the exam can be performed with the subject awake, i.e. in older children, using insert earphones or in free field, making the test more widely applicable and attractive.^7^, ^8^ Because it is an exogenous potential, the P1 component is related to the detection of the acoustic stimulus in the primary auditory cortex and, because it is widely thought not to be affected by sleep, can be used with waking or sleeping subjects.^9^

The need for accurate, reliable assessments, coupled with the major technological developments in recent decades, has paved the way for progress in studies in this area. In Australia, The National Acoustic Laboratories (NAL), the research division of the statutory authority Australian Hearing developed a device to record Cortical Auditory Evoked Potentials (CAEPs) and a protocol to objectively analyze the responses called the HearLab System, which has high sensitivity for detecting responses, reducing noise and artifacts. In addition, the detection of responses is performed in an automated fashion by the device, thereby reducing examiner subjectivity. In early latency responses, the evoked potentials are relatively stable, but in late latency evoked potentials, detection can be impaired by the instability of the true evoked potential, as well as residual noise.^10^ CAEPs in young children show more variability than those of adults because of increased electrophysiological noise.^11^ As a result, the common method for response detection, with visual observation of response, a plausible latency for key response components and response tracking (i.e., increased latency and decreased amplitude of the response with decreasing stimulus presentation levels) may be inadequate in this population. So, methods that reduce residual noise, such as those present in the HearLab System, are needed.^10^

Some authors have shown that automated response detection can make a valuable contribution in exploring these potentials in the infant population, but few have reported their efficacy in establishing thresholds.^12^, ^13^, ^14^, ^15^

This study is particularly relevant in the current context of infant audiology assessment as no studies using automated CAEP analyzers or standardizing responses in the neonatal population are available.

Therefore, our objective is to analyze the CAEP in terms of latencies, amplitudes at 80 dB HL and thresholds of CAEP at the frequencies 500, 1000, 2000 and 4000 Hz using an automatic response detection device in neonates with presence of otoacoustic emissions and no risk factors for hearing loss.

## Methods

The method was submitted to and approved by the Research Ethics Committee of Santa Casa de Sao Paulo (n° 951.829). The parents of the participants were informed about the study objectives and, having agreed to the neonates taking part, freely signed an informed consent form.

The subjects included in the study were neonates recruited from the maternity ward between February and September 2015. The families were invited to take part in the study during their stay in the accommodation unit attached to the maternity ward. Neonates with up to 28 days of life that passed the Neonatal Auditory Screening with a gestational age of at least 37 weeks and no risk indicators for hearing loss, according to the guidelines of the Joint Committee on Infant Hearing,^16^ were selected.

Neonates that had neurological syndromes or abnormalities, pre- and perinatal complications, or extreme agitation and excess movement during the exam precluding completion of the assessment were excluded.

Initially, 45 neonates were selected to take part, six of which were subsequently excluded for not meeting the study inclusion criteria. This gave a final study sample of 39 neonates, comprising 19 females and 20 males.

CAEPs were recorded in response to pure-tone stimuli with frequencies of 500, 1000, 2000 and 4000 Hz presented in an intensity range between 0 and 80 dB HL using a single channel recording, with insert earphone ER-3A, alternating polarity, and an inter-stimulus interval of 1.125 ms.

Prior to study commencement, the device was calibrated in dB HL according to the technical criteria established by the manufacturer.

An otoscope was used to inspect the external acoustic meatus of the subjects assessed to exclude excess earwax and ensure that there were no contra-indications for use of insert earphones.

Disposable type electrodes were affixed at Fpz (ground), Cz (active) and M1 or M2 (reference) positions and the impedance was kept at a level under 5 kohms.

Exams were performed in a sound-proofed room with the subjects placed in the mother's lap or in an appropriate chair for the age of the infant. During the assessment, newborns remained in light sleep. The behavioral state of the neonates was monitored by two examiner audiologists, who monitored the newborns throughout the evaluation. Because it is an exogenous potential, the P1 component is related to the detection of the acoustic stimulus in the primary auditory cortex and, most studies agree that it has little relation to the stages of sleep or wakefulness.^9^ Therefore, in the present study neonates remained in light sleep.

Differential analog amplification was 1210 times using a 12 dB high-pass filter with octaves of 4000 Hz and a 6 dB low-pass filter with octaves below 3000 Hz. The tone burst was 40 ms, with alternating polarity, a cosine envelope, a 0.5 Hz stimulus speed, a rise-fall of 10 ms, and a plateau of 30 ms. The rejection of artifacts was based on the current difference of the active-reference electrodes established by the device. Responses present with a minimum of 50 stimuli delivered were accepted when the objective detection statistic was *p* < 0.001. Otherwise, a CAEP response was judged to be present if the *p*-value reached the level of *p* < 0.05 after reaching the accepted number of 150 epochs. Residual noise was controlled during all assessments, and the HearLab display indicated the quality of the cortical response recorded in relation to the noise level of the signal. A residual noise level value less or equal to 3.2 μV indicates a good quality recording; a value between 3.2 and 3.6 μV indicates a slightly compromised recording and a value higher than 3.6 μV indicates a poor quality recording. In this study, the maximum value allowed for noise was 3.6 μV and for this reason participants with extreme agitation and excess movement were excluded. The ambient noise level did not exceed 35 dB SPL.

Physiological assessment was performed by studying cortical auditory evoked potentials by monaural acoustic stimulation, with the test ear chosen randomly. A total of 15 right ears and 25 left ears were tested at frequencies of 500, 1000, 2000 and 4000 Hz with amplitudes, latencies and electrophysiological thresholds recorded for each frequency assessed.

The stimuli were presented, with minor modifications, according to the acoustic stimuli decision protocol proposed by Van Dun et al.^17^ for greater ease-of-use and speed in CAEP responses. The initial maximum intensity was set at 80 dB HL to assess the integrity of the auditory pathway at the central level. Subsequently, the stimulus was tested at an intensity of 30 dB HL. In the absence of response at this intensity, the stimulus was increased in 5 dB increments until the threshold was detected. In the event of a response at 30 dB HL, the stimulus was delivered at an intensity of 15 dB HL, 5 dB HL and 0 dB HL. In the absence of response at 5 dB HL, the stimulus was increased in 5 dB HL increments until the electrophysiologic threshold was attained. All subjects were tested using this modified protocol. The stimuli in the different frequencies were presented randomly in order to avoid the habituation of the central auditory nervous system.^7^

The presence of P1 was objectively tested using Hotelling's *T*^2^ test, a multivariate extension of the ordinary one-sample *t*-test. Hotelling's *T*^2^ test takes vector data and tests a null hypothesis that the true mean vector equals a specified vector, in this case the zero vector. Each “data point” was a nine-dimensional binned epoch, and the null hypothesis being tested was that the averaged cortical response in every bin was zero, which considers responses present when *p* < 0.05. This method has shown great sensitivity and specificity in the detection of cortical responses.^8^ When there were high levels of noise or doubts about the P1 responses, the assessment was performed more than once.

The recording of the latency and amplitude of the P1 responses at 80 dB HL intensity was performed by three researchers with experience in electrophysiology, since the equipment does not automatically do this. P1 was considered in the highest peak in latency of 100–500 ms.

The amplitude and latency were considered only at 80 dB HL since the objective of the present study was to verify the speed and recruitment of neurons of the auditory cortex at a strong intensity. In addition, at lower intensities the latency and amplitude may be variable, which could compromise the comparison between the groups.

**Figure 1:**
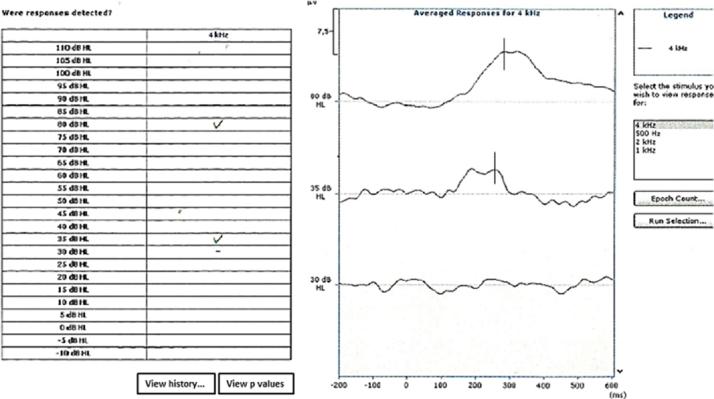
An example of automatic cortical auditory evoked potential threshold estimation to 4000 Hz. In the example, the equipment considered response at 80 and 35 dB HL. The black continuous line indicates the P1 latency considered by the three examiners.

Fig. 1 shows an example of an automated cortical auditory evoked potential threshold estimates in a neonate.

The Mann–Whitney, Wilcoxon and Repeated-Measures Analysis of Variance (ANOVA) tests were used for statistical analysis. In all tests, a significance level of 0.05 (or 5%) was adopted for rejection of the null hypothesis.

## Results

Average duration of an exam was 73.3 min, with the shortest being 38 min and the longest 111 min. This duration varied not only due to the responses but also as a result of movement and agitation of the subjects.

Latency and amplitude values at 80 dB HL intensity for the frequencies tested are given in Table 1, Table 2, respectively, showing no statistically significant difference among frequencies.

**Table 1:** P1 latency values (ms) at 80 dB HL for the frequencies tested.

P1 latency	500 Hz	1000 Hz	2000 Hz	4000 Hz	*p*-value
Mean	242.79	225.54	232.74	244.51	
Median	243	226	231	241	
Standard deviation	51.30	36.31	39.84	46.26	0.411
Minimum	137	125	160	157	
Maximum	419	307	353	370	
CI	33.27	23.54	25.83	30	
*N*	39	39	39	39	

CI, confidence interval; *N*, number of subjects.

**Table 2:** P1 amplitude values (μV) at 80 dB HL for the frequencies tested.

P1 amplitude	500 Hz	1000 Hz	2000 Hz	4000 Hz	*p*-value
Mean	6.41	7.36	6.31	5.88	
Median	6.07	7.15	5.43	5.78	
Standard deviation	3.39	4.10	3.31	2.83	0.550
Minimum	1.30	2.48	2.13	1.94	
Maximum	18.78	21.83	14.49	15.95	
CI	2.2	2.66	2.14	1.84	
*N*	39	39	39	39	

CI, confidence interval; *N*, number of subjects.

The mean threshold obtained was 24.8 ± 10.4 dB HL, 25 ± 9.0 dB HL, 28.72 ± 7.84 dB HL and 29.4 ± 6.6 dB HL for 500, 1000, 2000 and 4000 Hz, respectively. No statistically significant differences among the frequencies tested were found (Table 3).

**Table 3:** Description of cortical thresholds for the frequencies tested (dB HL).

Thresholds	500 Hz	1000 Hz	2000 Hz	4000 Hz	*p*-value
Mean	24.87	25	28.72	29.49	
Median	25	30	30	30	
Standard deviation	10.41	9.03	7.84	6.66	0.085
Minimum	0	0	0	15	
Maximum	40	35	40	50	
CI	6.76	5.86	5.08	4.32	
*N*	39	39	39	39	

CI, confidence interval; *N*, number of subjects.

**Figure 2:**
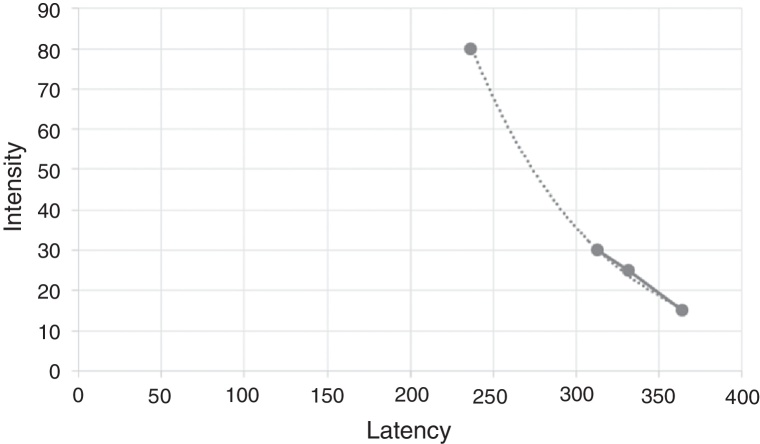
Representation of the latency function of the cortical potential P1 by intensity.

Fig. 2 shows the latency as a function of intensity, being the latency of the component P1 inversely proportional to the intensity of the acoustic stimulus.

## Discussion

The need to increase knowledge on hearing in the first months of life has prompted researchers to dedicate a great deal of attention to the search for new procedures in this area. Objective methods of assessing hearing have proven effective for this purpose, particularly in neonates and young infants. With the technical advances of recent decades, CAEP performed by automated devices has gained importance in this field. However, the paucity of studies using this method and the absence of parameters in the infant population prompted the present study.

The effectiveness of automated statistical detection compared to experienced examiners in detecting the presence of infant CAEP has already been studied in other research, evidencing its reliability.^10^ The protocol devised in this study for assessing hearing in neonates using a device for automatic response detection of CAEP, according to the modifications applied to the Van Dun model,^17^ proved effective and viable in detecting cortical responses in 100% of the subjects tested. The initial study at 80 dB HL allowed determination of P1 latency and amplitude in the group assessed, enabling analysis of the processing of sound stimuli at the central level.

Some audiologists have difficulties in detecting and interpreting electrophysiological responses based exclusively on visual analysis of responses, with such methods relying on subjectivity and the clinical experience of the examiner, particularly at levels that are low or border thresholds.^8^, ^15^ The automatic device for CAEP response detection and the statistical method adopted by the device proved sensitive and reliable for detecting cortical responses, corroborating the results of previous studies.^7^, ^8^, ^10^

Studies of cortical thresholds have revealed a correlation between electrophysiological thresholds and behavioral methods of assessing hearing both in normal hearing and hearing-impaired populations.^13^, ^17^, ^18^

In the present study, normal hearing neonates were assessed at specific frequencies. Thresholds of 24.8, 25, 28.7 and 29.4 dB HL were found for the frequencies of 500, 1000, 2000 and 4000 Hz, respectively. No significant differences were observed among the frequencies tested, rendering this procedure attractive compared to other methods of establishing electrophysiological thresholds, since other studies have reported higher thresholds at lower frequencies of 500 Hz.^19^ In a meta-analysis, Stapells^20^ reports the thresholds for Brain Stem Auditory Evoked Potential in normal hearing children to be: 19.5 (± 0.5) dB HL at 500 Hz, 17.4 (± 0.7) dB HL at 1000 Hz, 13.6 (± 0.9) dB HL at 2000 Hz and 15.5 (± 0.7) dB HL at 4000 Hz. CAEP thresholds obtained in this study were higher than those describe by Stapells in Brain Stem Auditory Evoked Potentials.^20^ Further studies are required to confirm these findings, but the rapid maturation of the brainstem in comparison to the central nervous system may influence the thresholds obtained. One study^7^ reported that the corrections and standard deviations between the auditory cortical thresholds and the behavioral thresholds in adults were 17.2 ± 7.4 for 500 Hz, 15.5 ± 6.0 for 1000 Hz, 16.8 ± 7.8 for 2000 Hz and 16.0 ± 8.4 for 4000 Hz. These values can be subtracted from the thresholds found from a fully automated CAEP to estimate behavioral thresholds.

Although the average CAEP thresholds were below 30 dB HL, the variation of thresholds was 0 to 50 dB NA. This fact evidences and corroborates with the scientific literature, which shows that in some cases of normal hearing the cortical thresholds can be higher than behavioral thresholds.^10^ Cone and Whitaker^21^ evidenced the cortical auditory potential P1 in 30 dB HL in 77% of infants, demonstrating that the responses are more difficult to visualize in thresholds, related to the immaturity of central areas. Some researchers suggest that about 33% of CAEP responses may be absent even when the stimuli are audible^14^ and that in newborns and young children, the immature response can be recordable for stimuli well above threshold.^22^

The present analysis of latencies for the frequencies tested yielded values of 242.79 for 500 Hz; 225.54 for 1000 Hz; 232.74 for 2000 Hz and 244.51 for 4000 Hz in 80 dB HL. These findings mirror those reported by Sharma,^4^ who found a marked positive peak, P1, at around 200–300 ms after a sound stimulus in normal hearing neonates. In the present study, no statistically significant differences were observed among the frequencies, corroborating the results of Golding,^5^ who despite having used speech stimuli, also found no differences among stimuli. However, some authors suggest that sounds are decoded differently in the auditory córtex,^23^, ^24^ a finding not observed in the present study, possibly explained by the fact that only the ability to detect stimuli in the auditory cortex was shown. Some researches describe longer latencies for the tone burst stimuli when compared with speech sounds.^21^ Although speech stimuli better represent stimulus processing at the central level, it was not possible to compare speech stimuli with tone burst in the present study, since the module used in the equipment does not have speech sound. Regarding amplitudes, values were 6.41, 7.36, 6.31 and 5.88 μV for the frequencies 500, 1000, 2000 and 4000 Hz, respectively. No similar studies were found in the literature for comparison against these results, rendering them reference values for the protocol used.

In this study, there was an increase in latency as intensity decreased. This fact corroborates another study, which showed that like the other auditory evoked potentials, latency is influenced by acoustic intensity due to lower neuronal stimulation.^22^

Also, the study of CAEP can objectively contribute to increasing knowledge on the maturation process of the auditory pathway.^5^, ^6^, ^25^ The development of the hearing system commences during gestation and continues through to adolescence, from peripheral to central structures.^24^, ^26^ Recent studies on brainstem AEP using speech sounds^27^ have shown shifting pattern of responses of BAEP across different phases of the lifespan, establishing that the plasticity of brainstem development continues beyond the first two years of life. Future investigation of BAEP can help elucidate the process of plasticity and stability of the auditory pathways.

Assessment duration in the pediatric population is also a key factor for successful diagnosis. This study showed shorter times than other similar clinical procedures, with an average test time of 1 h 13 min, shorter than electrophysiological assessment by other methods.^19^ The average duration of the exam was 73.3 min. Other researches^10^ have described an average duration of the cortical recording session of around 43 min, while other researchers^7^ reported a time of 40 min in testing the cortical threshold of hearing impaired subjects. This difference can be explained by the fact that the researchers used different transducers to evoke the responses.

The results of this study have demonstrated the effectiveness of using automatic response detection when estimating cortical thresholds in an infant population. Despite the positive results of this study, further studies are needed to examine the concordance of the results of measuring cortical thresholds automatically, compared with behavioral assessments, both in normal hearing children and in those with hearing loss. Some studies^10^ have reported that cortical thresholds are more easily visualized in children with hearing loss due to the presence of recruitment, which increases the amplitude of responses. However, the same researchers affirm that Hotelling's *T*^2^ test, part of the HearLab System, can detect normal auditory thresholds in children.

Although some established procedures are widely used for assessing electrophysiological thresholds, such as Brain Stem Auditory Evoked Potential and the more recent Stable-State Auditory Evoked Potential, no single method should be analyzed alone. While objective techniques are needed in this age group, monitoring hearing should primarily entail behavioral audiology assessment. In this study, the correlation between cortical and behavioral assessment was not performed because of the limitations of behavioral assessment in children younger than six months.

CAEP allows determination, both at the age group assessed and also in individuals that cannot be assessed by the behavioral approach, of the way in which the sound stimulus reaches the auditory cortex, thereby enriching the objective hearing assessment protocol. Reliable responses were obtained in the assessment of cortical potentials in the neonates assessed with a device for automatic response detection.

## Conclusion

The method devised was effective for automated response analysis in estimating auditory thresholds of normal hearing neonates using specific frequency stimuli, with mean values of 24.8 dB HL for 500 Hz; 25 dB HL for 1000 Hz; 28.7 dB HL for 2000 Hz and 29.4 dB HL for 4000 Hz.

Latency and amplitude values at 80 dB HL intensity showed no statistically significant difference among the frequencies tested. The latency of the component P1 is inversely proportional to the intensity of the acoustic stimulus.

## Funding

The HEARLab System was purchased with research funds (FAPESP 2011/19556-3).

## Conflicts of interest

The authors declare no conflicts of interest.
